# Patient-Reported Outcomes of Regular Aerobic Exercise in Gastric Cancer

**DOI:** 10.3390/cancers13092080

**Published:** 2021-04-25

**Authors:** Myung-Kyung Lee, Jihyun Oh

**Affiliations:** 1 College of Nursing, Research Institute of Nursing Science, Kyungpook National University, Daegu 41944, Korea; mlee@knu.ac.kr; 2 Department of Nursing, Daejeon University, Daejeon 34520, Korea

**Keywords:** exercise, depression, quality of life, gastric cancer

## Abstract

**Simple Summary:**

The benefits of exercise for health-related quality of life (HRQOL) and depression in patients with gastric cancer remain unclear. Thus this research was suggested to examine the association between maintenance of regular aerobic exercise at a recommended level and depression and HRQOL in patients with gastric cancer during or after treatment. The findings indicated that exercise can have a synergistic effect on improvement in HRQOL via indirect positive effects on depression and direct effects on HRQOL in patients with gastric cancer. The findings of this research may add the evidence on the benefits of exercise for HRQOL and depression in patients with gastric cancer to the research community.

**Abstract:**

The benefits of exercise for health-related quality of life (HRQOL) and depression in patients with gastrointestinal disease remain unclear, and studies on gastric cancer are scant. This study examines the association between the maintenance of regular aerobic exercise at a recommended level and depression and HRQOL in patients with gastric cancer during or after treatment. In this cross-sectional study, a face-to-face survey was used to collect data from 126 patients with primary gastric cancer during or after treatment in a tertiary acute-care hospital in Korea. Regular exercise was defined as regularly maintained aerobic exercise of at least moderate intensity consuming ≥4 metabolic equivalents for ≥150 min/wk for at least 6 months. Depression was measured using the 9-item version of the Patient Health Questionnaire, and HRQOL was assessed using the EORTC QLQ-C30. Patients who maintained aerobic exercise at a recommended level were less likely to have depression and more likely to have improved global QOL, as well as physical, role, and emotional functioning. Patients with depression were less likely to report improved global QOL and functioning. Thus, exercise can have a synergistic effect on improvement in HRQOL via indirect positive effects on depression and direct effects on HRQOL in patients with gastric cancer. Oncology nurses should encourage patients with gastric cancer who suffer from a depressive mood to set up and implement specific plans for practicing regular exercise, which can lead to an improvement in both depression and HRQOL.

## 1. Introduction

Although the incidence of gastric cancer has been declining steadily, it remains one of the most frequent and fatal cancers worldwide [1]. In 2018, there were more than 1,000,000 new cases of gastric cancer and 783,000 deaths, which corresponds to the fifth-highest incidence among all cancers and the third leading cause of cancer-related deaths worldwide. Recently, the 5-year survival rate of patients with gastric cancer was estimated to be 67% [2]. With the high survival rate of patients with gastric cancer, maintaining a healthy lifestyle is becoming more important.

Physical exercise is a regular, repeated, and specific physical activity that an individual can engage in during their spare time with the objective of cardiorespiratory fitness, strength, or health [3]. The guidelines from the American Cancer Society recommend that cancer survivors engage in regular exercise for at least 150 min/wk [4]. Recent studies have reported that regular exercise is an effective strategy for improving physical fitness, cardiorespiratory fitness, pulmonary function, muscle mass and strength, health-related quality of life (HRQOL), fatigue, psychological status such as anxiety and depression, and sleep quality in breast, lung, head and neck, ovarian, colorectal, and lymphoma cancer survivors during or after treatment [5,6,7,8,9,10,11]. Studies of patients with gastric cancer during and after treatment have also shown the beneficial effects of exercise on HRQOL [12,13,14] and emotional [1], social, and role functioning [14].

Although many studies have reported the effects of exercise on HRQOL and psychological state, several recent studies have failed to identify the benefits of exercise. For example, exercise programs failed to significantly reduce depression and anxiety [5] and also failed to improve HRQOL [15] in patients with breast cancer undergoing chemotherapy. Moreover, researchers have suggested that the benefit of exercise is unclear for patients with gastrointestinal disease [16]. Exercise may cause nausea, reflux, abdominal cramps, and occasionally gastrointestinal bleeding [17]. Therefore, because of the inconsistent results regarding the effects of exercise on patients with cancer, and particularly, the potential negative influence on gastrointestinal disease, it is necessary to examine the relationship between exercise and both HRQOL and depression in patients with gastric cancer during and after treatment.

According to previous studies, in addition to exercise, factors that may cause a deterioration in HRQOL and depression in patients with gastric cancer include the presence of comorbidities [18,19,20], older age [21], being female [16,19,20], having a lower income [22], having a lower education level [23], and lack of a spouse [23,24]. Thus, these sociodemographic characteristics were considered as covariates to examine the association between exercise and both depression and HRQOL in this study.

The purpose of the current study was to examine the association between exercise maintenance at a recommended level for cancer survivors (i.e., regular exercise of at least 150 min/wk for at least 6 months) and both depression and HRQOL in patients with gastric cancer during or after treatment.

## 2. Methods

### 2.1. Study Design and Participants

A cross-sectional study was conducted on patients with gastric cancer treated in two provinces of tertiary education hospitals in South Korea during February 2021. The eligibility criteria were as follows: Patients aged ≥19 years, with a pathological diagnosis of primary gastric cancer (stages I, II, or III) within the previous 2 years, who had surgery as a primary treatment and who were undergoing or had completed other therapies, with no other history of cancer, who provided written consent to participate in the study, and patients who provided contact information for returning questionnaires. Patients taking a medication due to unstable mood (e.g., depressive or anxious mood), those with comorbidities that were possibly a contraindication to aerobic exercise according to international guidelines (i.e., uncontrollable diabetes mellitus with complications, chronic obstructive pulmonary disease, or advanced heart failure diagnosed within 1 year), those with physical or cognitive conditions contraindicated for exercise (i.e., cognition or sensorimotor dysfunction), those experiencing any side effects during exercise in daily life, and those who refused to participate were excluded.

No participants reported that they had experienced any side effects during exercise in daily life at the time of enrollment of the survey. During the survey, the recommended health and safety measures to help protect participants from COVID-19 were strictly enforced (using hand sanitizer, symptom checklist, face covering, fever check, ventilation, and cleaning and disinfecting properties). All procedures in this study involving human participants were performed in accordance with the Declaration of Helsinki. The Ethical Review Board of the Kyungpook National University approved the study protocol (approval no. KNU-2021-0016).

### 2.2. Data Collection

The research staff consecutively contacted those patients with gastric cancer who have visited outpatient clinics or self-help group meetings, and asked about their intention to participate in the study. Subsequently, eligibility criteria screening was performed using a screening checklist. Obtained written informed consent was obtained from patients after the research staff had explained the purpose, procedure, and the matters of cooperation for the study. After providing informed consent, each participant completed the self-reported questionnaire with research staff in a meeting room. Patients who were unable to complete the questionnaire immediately were offered the opportunity to complete it at home or in the admission ward, and they were asked to send it back in a return envelope with an attached stamp. To increase the response rate, participants were followed up with reminders. Patients with incomplete responses or missing questionnaires were contacted by research staff via telephone.

Quota sampling was used to match the study sample with the male/female incidence ratio of gastric cancer in South Korea (new male cases: 19,545 [66.9%]; new female cases: 9662 [33.1%]) [25]. A total of 155 patients who consecutively visited the outpatient clinic during February 2021 were initially eligible, among whom 124 (80%) patients agreed to participate, signed the informed consent form, and completed the questionnaire. The most common reasons for nonparticipation were privacy violation or time constraints.

We calculated the sample size using G*power 3.1.9.4 based on the following indices for multiple regression analysis or analysis: significance level = alpha = 0.05; effect size = 0.15 (small effect size); power = 80%; and number of predictors = 10. The minimum sample size was calculated as 118. Considering a 5% dropout rate, the final sample size was *n* = 124.

### 2.3. Measures

We used a self-reported, structured questionnaire to measure sociodemographic characteristics, comorbidity, regular exercise, depression, and HRQOL. With regard to sociodemographic characteristics, we recorded the patients’ sex, age, marital status, religious practice, current job status, monthly household income, residence, national health insurance type, number of family members, and private health insurance.

#### 2.3.1. Comorbidity

Comorbidity was measured using the modified Patient-Reported Charlson Comorbidity Index (PRO-CCI) questionnaire [26]. The modified PRO-CCI measures 18 morbidities or complications and is calculated by assigning a weight on the 18 morbidities or complications [26]. The score ranges from 0 to 21, with no comorbidity scored as 0 and the presence of one or more comorbidities or complications scored as ≥1.

#### 2.3.2. Regular Exercise

The practice of regular exercise was measured based on the 7-day exercise diaries in min per week. We defined practicing regular exercise as patients who regularly practiced aerobic exercise with at least moderate intensity that consumed at least 4 metabolic equivalents (3.5 mL O_2_/kg/min) for at least 150 min per week [4] for at least 6 months. The places for exercise and types of aerobic exercise were determined by individuals’ availability and their own choices within the individual environment. Aerobic exercise with at least moderate intensity such as brisk walking was recommended; thus, individuals could use treadmill in indoors, walking trail in outdoors, or climb hills in the neighborhood.

#### 2.3.3. Depression

Depression was measured using the 9-item version of the Patient Health Questionnaire (PHQ-9), which is a self-report form of the evaluation of depression in primary health care [27]. Each item was scored on a 4-point Likert-type scale as follows: 0 = *not at all*; 1 *= several days*; 2 = *more than half the days*; and 3 = *nearly every day*. After this, the circled numbers are summed to yield a total score ranging between 0 and 27. The interpretation of the total score is as follows: 1–4, minimal depression; 5–9, mild depression; 10–14, moderate depression; 15–19, moderately severe depression; and 20–27, severe depression [27]. The Korean versions of the PHQ-9 have been previously validated, with a reported Cronbach α of 0.88 [28]. In the present study, the Cronbach α was 0.86.

#### 2.3.4. HRQOL

HRQOL was assessed using the European Organization for Research and Treatment of Cancer (EORTC) Quality of Life Questionnaire C30 (QLQ-C30) [16]. The QLQ-C30 is an internationally validated, brief, self-reporting, 30-item questionnaire that assesses cancer-specific QOL. This questionnaire has five functional subscales (physical, social, role, cognitive, and emotional functioning), nine symptom subscales (fatigue, nausea/vomiting, pain, dyspnea, sleep disturbances, appetite loss, constipation, diarrhea, and financial impact), and a global QOL subscale. Among these subscales, the global QOL scale and five functional subscales were used to measure HRQOL. Each individual item was scored on a 4-point Likert-type scale (physical, social, role, cognitive, and emotional functioning) or a 7-point Likert-type scale (global QOL). Each linear-converted score ranged from 0 and 100, with a high score for the functional subscale and the global QOL subscale indicating better HRQOL. The Korean versions of the EORTC QLQ-C30 have been validated previously [29]. The Korean version of the instrument development study reported that the Cronbach α of the global QOL and four functional scales was >0.82, except for cognitive functioning at 0.60 [29]. In the present study, the Cronbach α for functional items and the global QOL and four functional scales was >0.78, with the exception of cognitive functioning, at 0.62. The feasibility and comprehensibility of the survey instrument were pretested with 15 gastric cancer survivors.

### 2.4. Statistical Analyses

Descriptive statistics were used to summarize sociodemographic characteristics. Using an independent *t*-test, we performed univariate analyses to examine the association of sociodemographic characteristics with depression and HRQOL. Using a χ^2^ test, we tested the association between sociodemographic characteristics and practicing exercise to determine the confounders.

For the multivariate logistic regression analyses, depression status was dichotomized as no depression (total PHQ-9 score = 0) and depression (total PHQ-9 score ≥1) [26], and the better HRQOL group was defined as global HRQOL on a functioning scale as a score of ≥66 [30]. We examined the association between depression and HRQOL using multivariate logistic regression analyses. Finally, we identified the association between maintaining regular exercise and both depression and HRQOL using multivariate logistic regression analyses. In the multivariate logistic regression analyses, we adjusted for variables that were significant in the univariate analyses and variables that were referenced in the literature on associated factors with HRQOL in patients with cancer [18,19,22,23].

Statistical analyses were two-sided, and *p*-values < 0.05 were considered statistically significant. All statistical analyses were performed using SAS 9.4 statistical programs (SAS Institute, Cary, NC, USA).

## 3. Results

### 3.1. Participant Characteristics

More than half of the patients were <65 years (61%), had a spouse (77%), were unemployed (61%), earned < $2000 per month (61%), and lived in a metropolitan area (76%) (Table 1).

### 3.2. Univariate Analyses: Association between Sociodemographic Characteristics and Both Depression and HRQOL

Women had more severe depression (*p* = 0.013) and lower physical (*p* = 0.001), role (*p* = 0.003), emotional (*p* = 0.002), cognitive (*p* = 0.018), and social functioning (*p* = 0.024) than men. Having a job was associated with higher physical (*p* = 0.014) and social functioning (*p* = 0.017). Higher income was associated with higher global QOL (*p* = 0.006) and physical functioning (*p* = 0.029). Living in a metropolitan area was associated with less depression (*p* = 0.025) and higher global QOL (*p* = 0.040) and physical (*p* = 0.006), role (*p* = 0.002), emotional (*p* = 0.027), cognitive (*p* = 0.006), and social functioning (*p* = 0.009). Having private health insurance was associated with higher social functioning (*p* = 0.048). A higher comorbidity index score was associated with lower physical (*p* = 0.048) and role functioning (*p* = 0.024) (Table 1).

### 3.3. Univariate Analyses: Association between Sociodemographic Characteristics and Performing Exercise

No sociodemographic characteristics were associated with maintaining regular aerobic exercise at a recommended level (Table 2).

### 3.4. Multivariate Analyses: Association between Depression and HRQOL

Multivariate analyses showed that patients who had minimal-to-severe depression were less likely to report a high global QOL (adjusted odds ratio [aOR] [95% confidence interval (CI)] = 0.34 [0.14–0.83], *p* = 0.019) and role (aOR [95% CI] = 0.25 [0.08–0.82], *p* = 0.022), emotional (aOR [95% CI] = 0.21 [0.07–0.65], *p* = 0.007), and social functioning (aOR [95% CI] = 0.04 [0.01–0.22], *p* < 0.001) (Table 3).

### 3.5. Multivariate Analyses: Association between Performing Regular Exercise and Both Depression and HRQOL

Multivariate analyses showed that patients who maintained aerobic exercise at a recommended level were less likely to have depression (aOR [95% CI] = 0.27 [0.10–0.77], *p* = 0.014) and more likely to have improved global QOL (aOR [95% CI] = 3.37 [1.43–7.96], *p* = 0.006) and physical (aOR [95% CI] = 7.21 [1.23–42.17], *p* = 0.028), role (aOR [95% CI] = 4.89 [1.07–22.26], *p* = 0.040), and emotional functioning (aOR [95% CI] = 7.23 [1.42–36.79], *p* = 0.017) (Table 4).

## 4. Discussion

This cross-sectional study was conducted to examine whether maintaining regular exercise in patients with gastric cancer positively affects their depression and HRQOL. The novel finding of the study was that patients with gastric cancer who maintained exercise regularly for at least 6 months had significantly improved depression and HRQOL than patients who did not exercise at the recommended level.

To the best of our knowledge, this is the first study to examine whether patients with gastric cancer and depression had significantly decreased HRQOL. The results indicate that these patients had a significant decline in global QOL and role, emotional, and social functioning compared to those without depression. Thus, if the patients’ depression was to improve, their global QOL and role, emotional, and social functioning could also be improved. Although few studies have examined the relationship between depression and HRQOL in patients with gastric cancer, the findings of previous studies on other types of cancer have supported the negative association between depression and HRQOL. For example, among patients with esophageal cancer [21], prostate cancer [31], gynecological cancer [24], hematological cancer [32], and breast cancer undergoing chemotherapy [33], depression was identified as an independent risk factor for reducing various aspects of HRQOL. Depression is characterized by feelings of sadness and can result in severe impairments that interfere with or limit one’s ability to carry out major life activities, both at work and at home [34]. Emotional functioning includes awareness, expression, and regulation of emotions. Moreover, role functioning refers to involvement in life situations related to family life, partner relationship, household chores, work for pay, studies, social life (including interactions with friends), leisure time activities, community involvement (including volunteer work), and everyday living activities [35]. Social functioning is defined as the level at which an individual functions in his or her social context, with such functions ranging between self-preservation and basic living skills to relationships with others in society [36]. From the definitions of the functioning aspects in HRQOL, it is not surprising that patients with depression may experience a deterioration in emotional, role, and social functioning.

The main findings of this study are that depression in patients with gastric cancer might be improved by practicing sustained regular aerobic exercise, which may also improve global QOL and physical, role, and emotional functioning. Many patients with cancer suffer from depression [37], which may interfere with their ability to cope with the burden of the cancer, result in treatment refusal, extend hospitalization, deteriorate quality of life, and increase suicide risk [38,39,40]. The prevalence of depression in patients with cancer ranged from 8% to 24% according to one meta-analysis [41]. Although few previous studies have examined the relationship between exercise and depression in patients with gastric cancer, prior studies on other types of cancers supported our main findings. For example, among long-term survivors of testicular cancer, the prevalence of depression was higher among those who were physically inactive than among those who were physically active [42]. Moreover, several studies on patients with breast cancer reported that participants who did not participate in physical activity were more depressed [43], and that moderate and vigorous levels of physical activity after chemotherapy reduced the level of depression [44]. The reason for the relationship between practicing sustained regular exercise and low depression might be that regular exercise can positively impact serotonin levels in the brain [45]. Indeed, increasing the levels of serotonin boosts the mood and the overall sense of well-being [44]. Exercise can also help improve patients’ appetite [46] and sleep quality [47], both of which are negatively affected by depression.

The mechanisms underlying antidepressant effects of exercise are unclear [48]. Several reliable physiological and psychological mechanisms have been reported [48] such as thermogenic hypothesis [49], endorphin hypothesis [50,51], monoamine hypothesis [52,53], distraction hypothesis [54], and self-efficacy improvement [55,56]. The thermogenic hypothesis suggests that elevated core body temperature following exercise is responsible for the reduction of depression symptoms [49]. The endorphin hypothesis predicts that exercise has a positive effect on depression due to an increased release of β-endorphins following exercise [50,51]. The monoamine hypothesis indicates that exercise leads to an increase in the availability of brain neurotransmitters (e.g., serotonin, dopamine, and norepinephrine) that are diminished in depression cases [52,53]. The distraction hypothesis suggests that physical activity serves as a distraction from worries and depressing thoughts [54]. The enhancement of self-efficacy through exercise involvement may be another way in which exercise exerts its antidepressant effects [55,56].

Given that this is a cross-sectional study, the negative association between exercise and depression could be because patients with depression are less likely to participate in exercise. According to a previous study, women with elevated depressive symptoms found exercise to be more difficult than those without depression [57]. Indeed, depression might make exercise feel more demanding, which could ultimately decrease the patients’ likelihood of engaging in regular exercise. However, previous experimental studies have supported a causal relationship between exercise and a reduction in depression [6,58].

Multidisciplinary care for depression includes the use of antidepressants [54,59], psychological therapy [60,61], nutritional intervention [62,63], and physical activity (particularly aerobic exercise) [64]. Their benefits have been reportedly limited [65], whereas psychological therapies such as cognitive behavioral therapy and interpersonal psychotherapy have been shown to be as effective as antidepressant medications [60,61]. Nutritional intervention is also hypothesized as a potential tool for preventing or treating depression as it is associated with a decrease in nutrient stores and systemic inflammatory markers related to depression [62,63]. The practice of physical activity, particularly aerobic exercise, has been reported as a potent antidepressant due to its anti-inflammatory action [64,66]. In this study, the effect of exercise on depression was once again identified.

The findings of the current study, namely, that practicing sustained regular exercise might help improve global QOL [10,12,13,14] and physical [38], role [14], and emotional [10,12,38] functioning among patients with gastric cancer, were consistent with prior experimental studies on various other types of cancer. In particular, prior studies on patients with gastric cancer who reported exercise intervention after surgery or during chemotherapy showed an improved HRQOL [12,13,14] and emotional [12], social, and role functioning [14], which supports the current finding. However, a previous study on patients with advanced lung cancer reported no significant effect of exercise on HRQOL or functional status [67]. Although most experimental studies have concluded that regular exercise has a positive influence on HRQOL in patients with cancer, there was some heterogeneity among the previous studies. The paucity of consistent evidence on the relationship between sustained regular exercise and HRQOL leaves room for further longitudinal and experimental studies on patients with gastric cancer.

Oncology nurses should encourage patients with gastric cancer who have a depressive mood throughout treatment to set up and implement specific plans for practicing regular exercise in their daily lives, which can lead to an improvement in patients’ depression and HRQOL. If oncology nurses can help patients to set gradual goals to improve their confidence in exercise and experience success in achieving exercise goals, patients may find it easier to sustain regular exercise.

Several limitations should be considered when interpreting the current results. One limitation of this study is its cross-sectional nature, and the fact that a causal relationship of whether sustained regular exercise practice causes a positive change in depression and HRQOL has not been clearly identified. However, regular exercise was measured with 7-day exercise diaries, a characteristic of a prospective study. This study tried to reinforce the quality of data on exercise using 7-day exercise diaries. Internal and external validity could still be threatened with its cross-sectional study design. Positive effects of aerobic exercise on depression and HRQOL from this study of patients with gastric cancer are not yet confirmed. Further experimental studies with a control group are required.

Despite some limitations, the strength of this study is that because of insufficient studies on the relationship between exercise and depression and HRQOL in patients with gastric cancer, this study might be valuable and its results provide basic information for future experimental studies on gastric cancer. In addition, because the participant data were collected at one large acute care hospital in a metropolitan city in South Korea, the results of this study cannot be generalized to all patients with gastric cancer. However, the hospital has applied standardized treatment protocols for gastric cancer, which would minimize any variations in treatment. In addition, we attempted to reduce the sampling bias by using quota sampling to correct the bias, which might have been caused by a sex ratio of gastric cancer incidence.

## 5. Conclusions

Depression can be improved by sustained regular exercise, and this improvement in depression can lead to an improvement in HRQOL. In addition, sustained regular exercise itself directly contributes to an improvement in HRQOL. Therefore, exercise can have a synergistic effect on improving HRQOL by indirectly affecting depression and by directly affecting HRQOL in patients with gastric cancer. Further experimental studies with a control group should be conducted to examine the causal relationship between sustained regular exercise and HRQOL in patients with gastric cancer with or without depression. Additionally, oncology nurses play an essential role in assisting patients to perform at least moderate physical activity and encouraging patients to participate in exercise intervention programs. Therefore, exercise intervention programs should be developed to facilitate physical activity engagement because patients with cancer often get depressed and may have less motivation.

## Figures and Tables

**Table 1:** Univariate analyses: association between sociodemographic characteristics and depression and health-related quality of life.

Sociodemographic Characteristics	*n* = 124	PHQ Total Score	QL	PF	RF	EF	CF	SF
	*n* (%)	M (SD)	M (SD)	M (SD)	M (SD)	M (SD)	M (SD)	M (SD)
Sex								
Male	83 (66.9)	3.4 (4.3)	63.7 (21.9)	81.9 (16.5)	85.6 (22.0)	85.4 (17.2)	88.9 (13.8)	82.3 (19.2)
Female	41 (33.1)	5.6 (5.0)	56.5 (22.5)	71.2 (17.9)	72.4 (25.2)	72.9 (25.5)	80.9 (18.8)	72.5 (27.9)
*p*-value		0.013	0.093	0.001	0.003	0.002	0.018	0.024
Age (years)								
<65	76 (61.3)	4.4 (4.9)	62.0 (22.2)	79.9 (16.6)	82.7 (21.8)	82.1 (19.5)	87.5 (15.9)	78.4 (24.5)
≥65	48 (38.7)	3.8 (4.4)	60.2 (22.7)	75.8 (19.1)	78.8 (26.8)	80.2 (23.4)	84.4 (16.1)	80.2 (19.9)
*p*-value		0.533	0.663	0.213	0.385	0.626	0.279	0.677
Marital status								
No spouse	29 (23.4)	3.3 (4.2)	58.3 (25.2)	79.5 (19.4)	76.8 (28.5)	81.2 (25.4)	85.1 (19.9)	81.5 (20.8)
With spouse	95 (76.6)	4.4 (4.8)	62.2 (21.5)	78.0 (17.2)	82.5 (22.3)	81.4 (19.8)	86.6 (14.8)	78.5 (23.3)
*p*		0.240	0.428	0.683	0.271	0.957	0.663	0.546
Practice a religion								
No	45 (36.3)	3.8 (4.4)	59.4 (23.3)	81.5 (16.8)	85.5 (21.2)	83.3 (19.3)	88.0 (14.3)	79.0 (23.7)
Yes	79 (63.7)	4.3 (4.8)	62.4 (21.8)	76.5 (17.9)	78.6 (25.0)	80.2 (22.0)	85.3 (16.9)	79.2 (22.3)
*p*		0.539	0.476	0.132	0.122	0.425	0.352	0.956
Current job status								
No	75 (60.5)	4.4 (4.8)	58.7 (23.5)	75.3 (18.9)	79.1 (26.0)	79.2 (22.9)	85.1 (16.2)	75.1 (23.9)
Yes	49 (39.5)	3.7 (4.5)	65.2 (20.0)	82.8 (14.5)	84.4 (19.9)	84.5 (17.6)	88.0 (15.8)	85.0 (19.7)
*p*-value		0.434	0.112	0.014	0.229	0.173	0.332	0.017
Monthly household income (US$)								
<2000	75 (60.5)	4.1 (4.8)	56.9 (21.5)	75.5 (18.2)	79.1 (26.5)	78.6 (22.5)	85.1 (15.9)	77.5 (22.1)
≥2000	49 (39.5)	4.2 (4.5)	68.0 (22.1)	82.6 (16.0)	84.4 (19.1)	85.5 (18.0)	88.1 (16.3)	81.6 (23.6)
*p*-value		0.907	0.006	0.029	0.200	0.073	0.314	0.323
Residence								
Town, rural	30 (24.2)	5.8 (5.9)	55.3 (15.8)	68.7 (22.8)	66.7 (29.4)	73.9 (23.5)	77.2 (21.2)	69.5 (24.8)
Metropolitan	94 (75.8)	3.6 (4.1)	63.2 (23.8)	81.4 (14.5)	85.8 (19.8)	83.7 (19.7)	89.2 (12.9)	82.1 (21.3)
*p*-value		0.025	0.040	0.006	0.002	0.027	0.006	0.009
Health insurance								
National health insurance	100 (80.6)	4.1 (4.5)	60.9 (21.9)	79.3 (17.5)	82.0 (23.2)	82.5 (20.3)	86.9 (15.7	80.4 (22.1)
Medical aid	24 (19.4)	4.3 (5.3)	63.0 (24.2)	74.4 (18.0)	77.8 (26.8)	77.0 (23.6)	84.0 (17.7)	74.0 (25.0)
*p*-value		0.870	0.670	0.232	0.440	0.246	0.427	0.208
Number of family members								
0–1	35 (28.2)	4.1 (4.8)	61.1 (19.8)	75.2 (21.2)	77.1 (25.9)	78.3 (22.4)	81.0 (20.0)	77.1 (23.3)
≥2	89 (71.8)	4.2 (4.7)	61.4 (23.4)	79.5 (16.0)	82.8 (22.9)	82.6 (20.4)	88.4 (13.7)	79.9 (22.6)
*p*-value		0.928	0.955	0.224	0.240	0.315	0.046	0.543
Private health insurance								
No	69 (55.7)	4.3 (4.9)	57.9 (23.5)	77.5 (17.4)	79.7 (25.6)	79.2 (21.3)	86.0 (15.5)	75.5 (24.2)
Yes	55 (44.3)	3.9 (4.2)	65.6 (20.2)	79.4 (18.1)	83.0 (21.6)	84.1 (20.5)	86.7 (16.8)	83.6 (20.2)
*p*-value		0.678	0.054	0.546	0.438	0.198	0.817	0.048
Comorbidity index (modified PRO-CCI)								
0	68 (54.8)	3.8 (4.4)	60.1 (24.3)	81.2 (17.2)	85.6 (22.5)	84.5 (21.1)	87.6 (15.4)	79.8 (22.9)
≥1	56 (45.2)	4.6 (5.0)	62.7 (19.9)	74.9 (17.7)	75.9 (24.6)	77.8 (20.6)	84.8 (16.8)	78.4 (22.7)
*p*-value		0.323	0.513	0.048	0.024	0.078	0.341	0.729

Abbreviations: CF: cognitive functioning; EF: emotional functioning; M: mean; PF: physical functioning; PHQ: the Patient Health Questionnaire; QL: global quality of life (QOL); RF: role functioning; SD: standard deviation; SF: social functioning.

**Table 2:** Univariate analyses: association between sociodemographic characteristics and performing exercise.

	Aerobic Exercise with Moderate Intensity at Least 150 min/wk for at Least 6 Months (*n* = 124)		*p*
Sociodemographic Characteristics	No (*n* = 81 [65%]) *n* (%)	Yes (*n* = 43 [35%]) *n* (%)	
Sex			
Male	54 (66.7)	29 (67.4)	
Female	27 (33.3)	14 (32.6)	0.930
Age (years)			
<65	51 (63.0)	25 (58.1)	
≥65	30 (37.0)	18 (41.9)	0.599
Marital status			
No spouse	19 (23.5)	10 (23.3)	
With spouse	62 (76.5)	33 (76.7)	0.980
Practice a religion			
No	30 (37.0)	15 (34.9)	
Yes	51 (63.0)	28 (65.1)	0.812
Current job status			
No	52 (64.2)	23 (53.5)	
Yes	29 (35.8)	20 (46.5)	0.246
Monthly household income (US$)			
<2000	49 (60.5)	26 (60.5)	
≥2000	32 (39.5)	17 (39.5)	0.997
Residence			
Town, rural	23 (28.4)	7 (16.3)	
Metropolitan	58 (71.6)	36 (83.7)	0.134
Health insurance			
National health insurance	65 (80.3)	35 (81.4)	
Medical aid	16 (19.7)	8 (18.6)	0.878
Number of family members			
0–1	21 (25.9)	14 (32.6)	
≥2	60 (74.1)	29 (67.4)	0.435
Private health insurance			
No	49 (60.5)	20 (46.5)	
Yes	32 (39.5)	23 (53.5)	0.136
Comorbidity index (modified PRO-CCI Questionnaire)			
0	46 (56.8)	22 (51.2)	
≥1	35 (43.2)	21 (48.8)	0.549

Abbreviation: PRO-CCI: patient-reported Charlson Comorbidity Index.

**Table 3:** Multivariate analyses: association between depression status and health-related quality of life.

Dependent Variable	The Patient Health Questionnaire (*n* = 124)		*p*
	No Depression (*n* = 88 [71%])	Minimal to Severe Depression (*n* = 36 [29%])	
Global QOL			
Low (<66.66), *n* (%)	37 (42.1)	25 (69.4)	
High (≥66.66), *n* (%)	51 (57.9)	11 (30.6)	
aOR^a^ (95% CI) for higher QOL (≥66.66)	1 (reference)	0.34 (0.14–0.83)	0.019
Physical functioning (PF)			
Low (<66.66), *n* (%)	10 (11.4)	8 (22.2)	
High (≥66.66), *n* (%)	78 (88.6)	28 (77.8)	
aOR^a^ (95% CI) for higher PF (≥66.66)	1 (reference)	0.47 (0.15–1.51)	0.205
Role functioning (RF)			
Low (<66.66), *n* (%)	8 (9.1)	10 (27.8)	
High (≥66.66), *n* (%)	80 (90.9)	26 (72.2)	
aOR^a^ (95% CI) for higher RF (≥66.66)	1 (reference)	0.25 (0.08–0.82)	0.022
Emotional functioning (EF)			
Low (<66.66), *n* (%)	8 (9.1)	12 (33.3)	
High (≥66.66), *n* (%)	80 (90.9)	24 (66.7)	
aOR^a^ (95% CI) for higher EF (≥66.66)	1 (reference)	0.21 (0.07–0.65)	0.007
Cognitive functioning (CF)			
Low (<66.66), *n* (%)	5 (5.7)	5 (13.9)	
High (≥66.66), *n* (%)	83 (94.3)	31 (86.1)	
aOR^a^ (95% CI) for higher CF (≥66.66)	1 (reference)	0.47 (0.11–2.11)	0.327
Social functioning (SF)			
Low (<66.66), *n* (%)	7 (8.0)	15 (41.7)	
High (≥66.66), *n* (%)	81 (92.0)	21 (58.3)	
aOR^a^ (95% CI) for higher SF (≥66.66)	1 (reference)	0.04 (0.01–0.22)	<0.001

Abbreviations: aOR: adjusted odds ratio, CI: confidence interval. aOR^a^ (95% CI) was derived from multivariate logistic regression analyses adjusted for sex, age, practicing a religion, current job status, monthly household income, residence area, national health insurance type, number of family members living together, private health insurance, and comorbidity index.

**Table 4:** Multivariate analyses: association between performing regular exercise and both depression and quality of life.

	Aerobic Exercise with Moderate Intensity at Least 150 min/wk for at Least 6 Months		*p*
Dependent Variable	No (*n* = 81 [65%])	Yes (*n* = 43 [35%])	
The Patient Health Questionnaire			
No depression, *n* (%)	51 (63.0)	37 (86.1)	
Minimal to severe depression, *n* (%)	30 (37.0)	6 (13.9)	
aOR^a^ (95% CI) for mild to severe depression	1 (reference)	0.27 (0.10–0.77)	0.014
Global QOL			
Low (<66.66), *n* (%)	49 (60.5)	13 (30.2)	
High (≥66.66), *n* (%)	32 (39.5)	30 (69.8)	
aOR^a^ (95% CI) for higher QL (≥66.66)	1 (reference)	3.37 (1.43–7.96)	0.006
Physical functioning (PF)			
Low (<66.66), *n* (%)	16 (19.7)	2 (4.6)	
High (≥66.66), *n* (%)	65 (80.3)	41 (95.4)	
aOR^a^ (95% CI) for higher PF (≥66.66)	1 (reference)	7.21 (1.23–42.17)	0.028
Role functioning (RF)			
Low (<66.66), *n* (%)	15 (18.5)	3 (7.0)	
High (≥66.66), *n* (%)	66 (81.5)	40 (93.0)	
aOR^a^ (95% CI) for higher RF (≥66.66)	1 (reference)	4.89 (1.07–22.26)	0.040
Emotional functioning (EF)			
Low (<66.66), *n* (%)	18 (22.2)	2 (4.6)	
High (≥66.66), *n* (%)	63 (77.8)	41 (95.4)	
aOR^a^ (95% CI) for higher EF (≥66.66)	1 (reference)	7.23 (1.42–36.79)	0.017
Cognitive functioning (CF)			
Low (<66.66), *n* (%)	8 (9.9)	2 (4.6)	
High (≥66.66), *n* (%)	73 (90.1)	41 (95.4)	
aOR^a^ (95% CI) for higher CF (≥66.66)	1 (reference)	1.78 (0.28–11.16)	0.538
Social functioning (SF)			
Low (<66.66), *n* (%)	17 (23.0)	5 (11.6)	
High (≥66.66), *n* (%)	64 (79.0)	38 (88.4)	
aOR^a^ (95% CI) for higher SF (≥66.66)	1 (reference)	1.03 (0.27–4.01)	0.962

Abbreviations: aOR: adjusted odds ratio, CI: confidence interval. aOR^a^ (95% CI) was derived from multivariate logistic regression analyses adjusted for sex, age, practicing a religion, current job status, monthly household income, residence area, national health insurance type, number of family members living together, private health insurance, and comorbidity index.

## Data Availability

The data presented in this study are available on request from the corresponding author. The data are not publicly available due to privacy.
